# Water intake and obesity: By amount, timing, and perceived temperature of drinking water

**DOI:** 10.1371/journal.pone.0301373

**Published:** 2024-04-25

**Authors:** Jaewon Khil, Qiao-Yi Chen, Dong Hoon Lee, Kyung-Won Hong, NaNa Keum

**Affiliations:** 1 Department of Food Science and Biotechnology, Dongguk University, Goyang, South Korea; 2 Department of Sport Industry Studies, Yonsei University, Seoul, South Korea; 3 Department of Nutrition, Harvard T.H. Chan School of Public Health, Boston, MA, United States of America; 4 Theragen Health Co. Ltd., Seongnam, South Korea; Severance Hospital, Yonsei University College of Medicine, REPUBLIC OF KOREA

## Abstract

Water intake has been suggested to be associated with weight control, but evidence for optimal water intake in terms of amount, timing, and temperature is sparse. Additionally, genetic predisposition to obesity, which affects satiety and energy expenditure, might interact with water intake in regulating individual adiposity risk. We conducted a cross-sectional study recruiting 172 Korean adults. Information on water intake and lifestyle factors was collected through self-reported questionnaires, and height, weight, and waist circumference (WC) were measured by researchers. The oral buccal swab was performed for genotyping of *FTO* rs9939609, *MC4R* rs17782313, *BDNF* rs6265 and genetic risk of obesity was calculated. Linear regression was performed to estimate mean difference in body mass index (BMI) and WC by water intake and its 95% confidence interval (95% CI). As a sensitivity analysis, logistic regression was performed to estimate odds ratio (OR) of obesity/overweight (BMI of ≥23kg/m^2^; WC of ≥90cm for men and of ≥80cm for women) and its 95% CI. Drinking >1L/day was significantly associated with higher BMI (mean difference: 0.90, 95% CI 0.09, 1.72) and WC (mean difference: 3.01, 95% CI 0.62, 5.41) compared with drinking ≤1L/day. Independent of total water intake, drinking before bedtime was significantly associated with lower BMI (mean difference: -0.98, 95% CI -1.91, -0.05). The results remained consistent when continuous BMI and WC were analyzed as categorical outcomes. By perceived temperature, drinking >1L/day of cold water was associated with higher BMI and WC compared with drinking ≤1L/day of water at room-temperature. By genetic predisposition to obesity, a positive association between water intake and WC was confined to participants with low genetic risk of obesity (P interaction = 0.04). In conclusion, amount, timing, and perceived temperature of water intake may be associated with adiposity risk and the associations might vary according to genetic predisposition to obesity.

## Introduction

The prevalence of obesity has reached epidemic proportions worldwide, with an estimated 58% of the world’s adult population predicted to become overweight or obese by 2030 [1]. To prevent weight gain or to achieve weight loss, it is generally advised to drink a plenty of water (e.g., “drink at least eight glasses of water a day”) along with decreasing calorie intake and increasing physical activity. However, neither the optimal amount of water intake for general health is established, nor evidence between water intake and obesity is consistent [2]. The discrepant results may be in part related to failures to account for timing of drinking or temperature of drinking water. For instance, pre-meal water intake, likely by increasing satiety, was associated with a reduced meal energy intake but post-meal water intake was not [3]. Although water intake during or after a meal has been claimed to interfere with digestion by diluting stomach acid and digestive enzymes, there is little evidence for the claim itself and its impact on body weight. Regarding water temperature, drinking cold water was reported to increase energy expenditure by requiring extra energy for heating up the water to body temperature [4], and to reduce energy intake by suppressing gastric motility [5]. Thus, addressing potential interaction of amount of water intake with timing of drinking and water temperature is critical to examining the relationship between water intake and adiposity.

Obesity, as a multifactorial disease, originates from interactions of multiple factors including genetic, environmental, and social factors. Candidates that might modify the effect of water intake on adiposity include single-nucleotide polymorphisms (SNPs) in obesity-associated genes including, representatively SNPs in the fat mass and obesity-associated gene (*FTO*) (rs9939609, T>A) [6–8], near the melanocortin 4 receptor gene (*MC4R*) (rs17782313, T>C) [9–11], in brain-derived neurotrophic factor gene (*BDNF*) (rs6265, C>T) [12, 13]. Previous studies have shown that these genetic variations are implicated in obesity risk by modulating energy intake and energy expenditure [14–16].

Considering that water intake affects satiety and energy metabolism, the aforementioned genetic variations and water intake may interact through the shared biological pathways in relation to obesity risk. Thus, in addition to examining the relationship between total daily water intake and adiposity risk accounting for timing of drinking and perceived water temperature, we also explored whether genetic predisposition to obesity as defined by *FTO* rs9939609, *MC4R* rs17782313, *BDNF* rs6265 modifies the relationship.

## Method

### Study participants

A cross-sectional study was conducted in Dongguk University, Goyang-si, Korea from March 2019 to May 2019. Participants were recruited through flyers posted at universities and nearby stores. We recruited healthy individuals aged 18 years or older. The exclusion criteria included the following conditions: with a history of major diseases or surgery, on regular treatment or prescription medicine, with mental illness or anemia, unable to exercise, or pregnant or planning for it. Initially, 180 individuals (119 men, and 61 women) visited the research center for questionnaire response, oral sampling, and anthropometric measurements. We further excluded 4 individuals with missing response on the amount of total daily water intake and 4 individuals with an outlier body mass index (BMI) (≥30kg/m^2^). The final analytic cohort for this study included a total of 172 individuals (75 men, 97 women).

All participants provided written informed consents and their participation in research was compensated with a coffee voucher and commercially available direct-to-consumer genetic testing were provided for participating in the study. In order to safeguard the personal information of participants, they were assigned anonymization codes. As a result, none of the researchers were able to identify the individual participants. This study was approved by the Dongguk University Ilsan Hospital Institutional Review Board (IRB number: 2018-12-006).

### Assessment of water intake and other covariates

A semi-quantitative frequency questionnaire was administered to collect information on water intake patterns (amount, timing) in the past year. The total daily water intake was measured by asking the number of glasses (500 ml) of water one drinks per day excluding tea/coffee/alcohol. Regarding the timing of water intake, participants were asked to report whether they drink on an empty stomach immediately after waking-up (yes, no) and before bed at night (yes, no). Water intake around a meal was assessed by asking for the usual number of glasses of water consumed (0, <1, 1–2, >2 glasses) at each of the three time points: 1) pre-meal (30 minutes before a meal), 2) intra-meal (during a meal), 3) post-meal (within 30 minutes after a meal). For measurement of perceived water temperature, participants answered the perceived temperature of the water they normally drink (at room temperature, warm, cold, any)

Using the frequency questionnaire, we also collected information on demographic factors, lifestyle, and dietary habits that could be potential confounders for the associations between water intake and adiposity risk. The list includes: age, sex, perceived temperature of drinking water, physical activity, perceived sleep deprivation, perceived stress level, smoking history, alcohol intake, sugar-sweetened beverage intake, breakfast intake, portion size of dinner, night-time snack intake.

### Genotyping of obesity-associated SNPs

With Korean government tightly regulating direct-to-consumer genetic testing service, the service was allowed to provide genetic information on the limited genes related to health and beauty [17]. In relation to obesity, genetic tests related to *FTO*, *MC4R*, *BDNF* genes were approved [17]. The direct-to-consumer genetic testing service that we provided to participants provided the genotypes of *FTO* rs9939609, *MC4R* rs17782313, *BDNF* rs6265. For this genetic testing, DNA was extracted from buccal swabs using ExgeneTM Tissue SV (GeneAll, Seoul, Korea) and genotypes of the SNPs were identified using a SNP array (Theragen PMRA, Seoul, Korea). The detailed procedures of genotyping and quality-control are provided elsewhere [18].

In an Asian population, the accuracy of the SNP array in genotyping was demonstrated to be 0.94 for SNPs with minor allele frequencies >5%. The minor allele frequency in this study population was 13% for *FTO* rs9939609 (T>A), 23% for *MC4R* rs17782313(T>C), 47% for *BDNF* rs6265 (C>T). The call rate≥97% and genotype distribution was in Hardy-Weinberg equilibrium (*χ*^2^ test, P>0.05)

Based on the three SNPs related to obesity, genetic predisposition to obesity was estimated. Participants were considered high genetic risk of obesity when they carry at least one risk allele of any of the three SNPs (A allele for FTO rs9939609 C allele for MC4R rs1778231and C allele for BDNF rs6265), but low genetic risk when they carry no risk allele at all.

### Ascertainment of obesity status

Height and weight of participants, who were in light clothing, were measured to the nearest 0.1 unit using an automatic stadiometer (BSM-330, Seoul, Korea) and a bioelectrical impedance analyzer (Inbody720, Seoul, Korea), respectively. BMI was calculated as weight in kilograms divided by the square of height in meters (kg/m^2^). Following the criteria for Asians set by the World Health Organization [19], the majority of our study population did not fall within the obese category, as indicated by the distribution of BMI (mean: 22.3, SD: 2.8 kg/m^2^) and waist circumference (WC) (mean: 76.0, SD: 8.8 cm) within the population. Thus, in our study, we defined overweight / obesity as a BMI ≥23kg/m^2^. This specific cut-off value accounts for the characteristic of Asians, who tend to have a higher proportion of fat mass for a given BMI when compared to other racial groups [19].

Waist circumference (WC) was measured midway between the lowest rib and the iliac crest in standing position, as recommended by the World Health Organization [20], to the nearest 0.1 cm using a flexible band. In accordance with the guidelines outlined by The Korean Society for the Study of Obesity [21], we defined central obesity as a WC of ≥90 cm for men and ≥80 cm for women.

### Statistical analysis

Characteristics of the participants were compared according to total daily water intake (≤1L/day, >1L/day) by using the χ2 test for categorical variables and t-test for continuous variables. The mean difference and 95% confidence interval (95% CI) form the linear regression and odd ratios and 95% CI from the logistic regression were used to examine the relationship between total daily water intake and adiposity (as indicated by BMI and WC), adjusting for potential confounders listed in the covariate assessment section. Of note, as water intake has been claimed to increase satiety and metabolic rate, overweight / obese subjects may intentionally increase their water intake as a weight loss effort. To check whether our results are driven by reverse causation, as a sensitivity analysis, we repeated the analysis within each subgroup defined using the medians of BMI and WC as the cutoffs.

To examine an effect of timing of water intake independent of the amount of daily water intake, linear regression was fitted using BMI and WC as dependent variables and water intake at different time points (immediately after waking-up in the morning, before bed at night, pre-, intra-, and post-meal) as independent variables, after adjusting for total daily intake and

To understand a combined effect of amount of water intake and perceived water temperature, we conducted linear regression analysis based on joint categories of water intake (≤1L/day, >1L/day) and perceived water temperature (room temperature, cold, any). Potential interaction between amount and perceived temperature of drinking water was evaluated by adding their product term in the regression model and by performing a Wald test on the interaction term.

To explore whether the relationship between total daily water intake and adiposity varies by genetic predisposition to obesity, we performed a stratified analysis by fitting a linear regression in each of subgroup of participants with low- and high- genetic risk of obesity as defined by a combination of *FTO* rs9939609, *MC4R* rs17782313, and *BDNF* rs6265. Heterogeneity in the relationship by genetic predisposition to obesity was evaluated by adding a product term between total daily water intake and genetic risk score in the regression model and performing a Wald test on the term.

P-value of <0.05 was considered statistically significant. All statistical analyses were performed using SAS 9.4 (SAS Institute, Cary, NC, USA).

## Result

### Baseline characteristics of participants by daily total water intake

Characteristics of the 172 recruited subjects are presented according to total daily water intake in Table 1. Approximately 53% of the participants drank ≤1L/day and 47% drank >1L/day. The range of BMI and WC in the study population was 15.9–29.5 kg/m^2^ and 58.0–100.0 cm respectively, with the means higher among participants drinking >1L/day. Compared with subjects who drank ≤1L/day, those who drank >1L / day were more likely to drink water immediately after waking-up in the morning, before bed at night, and before-, and after- meal. The two groups did not differ in the distribution of perceived temperature of drinking water. In addition, participants drinking >1L/day were more likely to be men, to engage in physician activity, and to smoke ≥100 cigarettes in lifetime.

**Table 1:** Characteristics of participants by total daily water intake.

	Total daily water intake (L/day)		P value
	≤ 1	>1	
Number of subjects, n	92	80	
BMI (kg/m^2^), mean (SD)	22 (2.6)	23 (2.7)	<0.01
WC (cm), mean (SD)	74 (8.5)	79 (8.3)	<0.01
Water intake immediately after waking up in the morning (%)			
No	52	12	<0.01
Yes	48	88	
Water intake before bed at night (%)			
No	37	14	<0.01
Yes	63	86	
Pre-meal water intake (%)			
< 1 cup/day	91	40	<0.01
≥ 1 cup/day	9	60	
Intra-meal water intake (%)			
< 1 cup/day	66	54	0.09
≥ 1 cup/day	34	46	
Post-meal water intake (%)			
< 1 cup/day	60	37	<0.01
≥ 1 cup/day	40	62	
Perceived water temperature			
Room temperature	27	29	0.95
Cold	59	56	
Any temperature	14	15	
Age, mean (SD)	22 (2.4)	22 (2.4)	0.77
Male sex (%)	28	61	<0.01
Physical activity (%)			
<1 hour/week	49	17	<0.01
1–3.9 hours/week	29	34	
≥4 hours/week	22	49	
Perceived sleep deprivation (%)			
No	45	41	0.66
Yes	55	59	
Perceived stress level (%)			
Hardly rare	8	16	0.17
Little bit	60	49	
A lot	38	26	
Very much	4	9	
Smoking history (%)			
< 100 cigarettes in lifetime	86	72	0.03
≥ 100 cigarettes in lifetime	14	27	
Alcohol intake (%)			
Never	39	45	0.74
1–2 times/week	47	42	
≥3 times/week	14	12	
Sugar-sweetened beverage intake (%)			
Never	25	24	0.21
0–1 time/week	33	45	
2–7 times/week	42	31	
Breakfast intake (%)			
Never	46	30	0.06
1–4 times/week	30	31	
5–7 times/week	24	39	
Portion size of dinner (%)			
Less full	4.6	10.9	0.41
Until not hungry	13.9	14.1	
Until slightly full	58.3	57.8	
Focuses on not leaving food	4.7	6.3	
Very full	18.5	10.9	
Night-time snack intake (%)			
Almost Never	36	36	0.14
1–2 times/week	43	31	
≥3 times/week	21	33	
Genetic predisposition to obesity (%)			
Low risk	16	9	0.14
High risk	84	91	

Abbreviation: SD, standard deviation; BMI, Body Mass Index; WC, waist circumference

Data are presented as mean (standard deviation) for continuous variable and proportion for categorical variables.

### Associations between water intake and adiposity by timing of intake

Compared to participants drinking ≤1L/day, those drinking >1L/day were significantly associated with 0.90 kg/m^2^ (95% CI 0.09, 1.72) higher BMI and 3.01 cm (95% CI 0.62, 5.41) higher WC, after adjustment for potential confounders (Table 2). The observed trends remained consistent after categorizing BMI and WC into obesity and normal (S1 Table). The pattern of higher BMI and WC being associated with participants drinking >1L/day water intake was consistently observed in all subgroups of ≤median BMI, >median BMI, ≤median WC, and >median WC (S1 and S2 Figs), rejecting reverse causality as a potential explanation for the observed associations.

**Table 2:** Linear regression coefficients for the associations between water intake and obesity by timing of water intake.

	BMI		WC	
	Univariable (95% CI) P-value	Multivariable* (95% CI) P-value	Univariable (95% CI) P-value	Multivariable* (95% CI) P-value
Total daily water intake (>1 vs. ≤1 L/day)	1.54 (0.73, 2.35), <0.01	0.90 (0.09, 1.72), 0.03	5.49 (2.95, 8.03), <0.01	3.01 (0.62, 5.41), 0.01
Drinking water immediately after waking up in the morning (Yes vs. No)**	-0.34 (-1.32, 0.65), 0.50	-0.26 (-1.16, 0.64), 0.57	0.30 (-2.81, 3.41), 0.85	0.66 (-2.01, 3.34), 0.63
Drinking water before bedtime (Yes vs. No)**	-0.85 (-1.84, 0.15), 0.10	-1.00 (-1.94, -0.07), 0.04	-2.09 (-5.23, 1.06), 0.19	-2.35 (-5.11, 0.42), 0.14
Pre-meal water intake (≥1 vs. <1 cup/meal)***	0.41 (-0.68, 1.5), 0.46	-0.29 (-1.35, 0.77), 0.59	3.53 (0.16, 6.90), 0.04	1.01 (-2.10, 4.11), 0.52
Intra-meal water intake (≥1 vs. <1 cup/meal)***	-0.21 (-1.06, 0.64), 0.62	-0.21 (-1.02, 0.60), 0.61	-0.54 (-3.18, 2.1), 0.69	-0.33 (-2.70, 2.05), 0.79
Post-meal water intake (≥1 vs. <1 cup/meal)***	0.03 (-0.85, 0.91), 0.94	-0.12 (-0.94, 0.70), 0.78	0.20 (-2.53, 2.92), 0.89	-0.71 (-3.11, 1.70), 0.56

Abbreviation: BMI, body mass index; WC, Waist Circumference

* adjusted for age, sex, genetic predisposition to obesity, perceived water temperature, physical activity, perceived sleep deprivation, perceived stress level, smoking history, alcohol intake, sugar-sweetened beverage intake, breakfast intake, portion size of dinner, night-time snack intake.

** additionally adjusted for total daily water intake; water intake immediately after waking-up and water intake before bedtime were mutually adjusted for

*** additionally adjusted for total daily water intake; pre-meal, intra-meal, and post-meal water intake were mutually adjusted for

Independent of total daily water intake, the effect of timing of water intake on adiposity was examined (Table 2). After controlling for total daily water intake and other confounders, drinking water immediately after waking-up was not associated with adiposity by BMI and WC, but drinking water before bedtime was associated with significantly lower BMI (mean difference: -1.00, 95% CI -1.94, -0.07) and non-significantly lower WC (mean difference: -2.35, 95% CI -5.11, 0.42) (Table 2). The observed trends remained consistent after categorizing BMI and WC into obesity and normal (S1 Table).

Regarding timing of water intake around a meal, after adjusting for total daily water intake and other confounders, none of pre-meal, intra-meal, and post-meal water intake was associated with adiposity as measured by BMI or WC.

### Joint associations of water intake and perceived water temperature with adiposity

**Fig 1:**
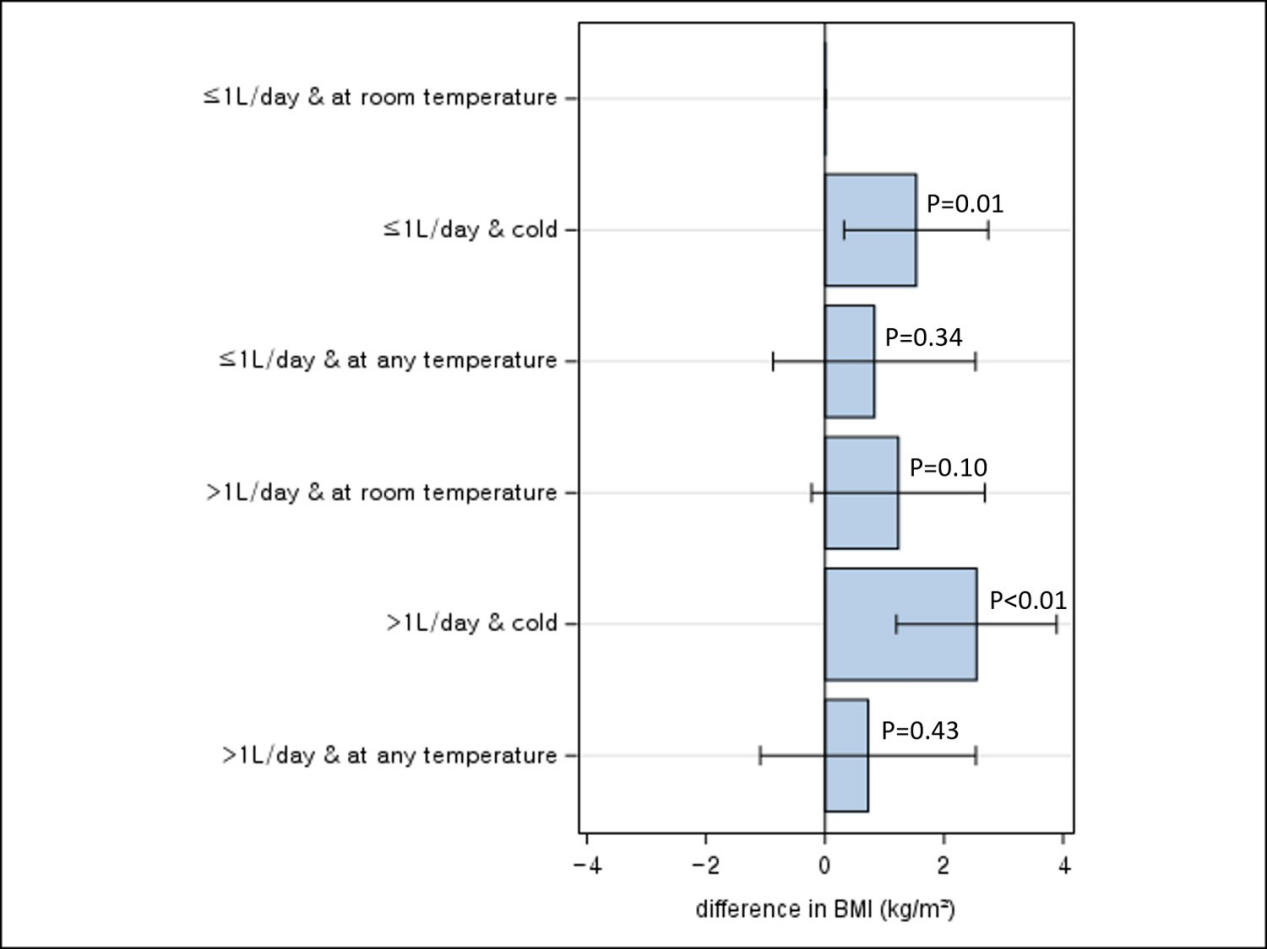
Joint association between total daily water intake and BMI according to perceived water temperature.

With BMI, compared to participants drinking ≤ 1L/day of water and preferentially room-temperature water, a significantly higher BMI was observed for those drinking ≤ 1L/day of water and preferentially cold water (mean difference: 1.53 kg/m^2^, 95% CI 0.31, 2.74) and for those drinking >1L/day and preferentially cold water (mean difference: 2.54 kg/m^2^, 95% CI 1.20, 3.89) (Fig 1). No significant difference in BMI was observed with other joint categories. The interaction between amount and perceived temperature of drinking water on BMI was not significant (P for interaction = 0.53).

**Fig 2:**
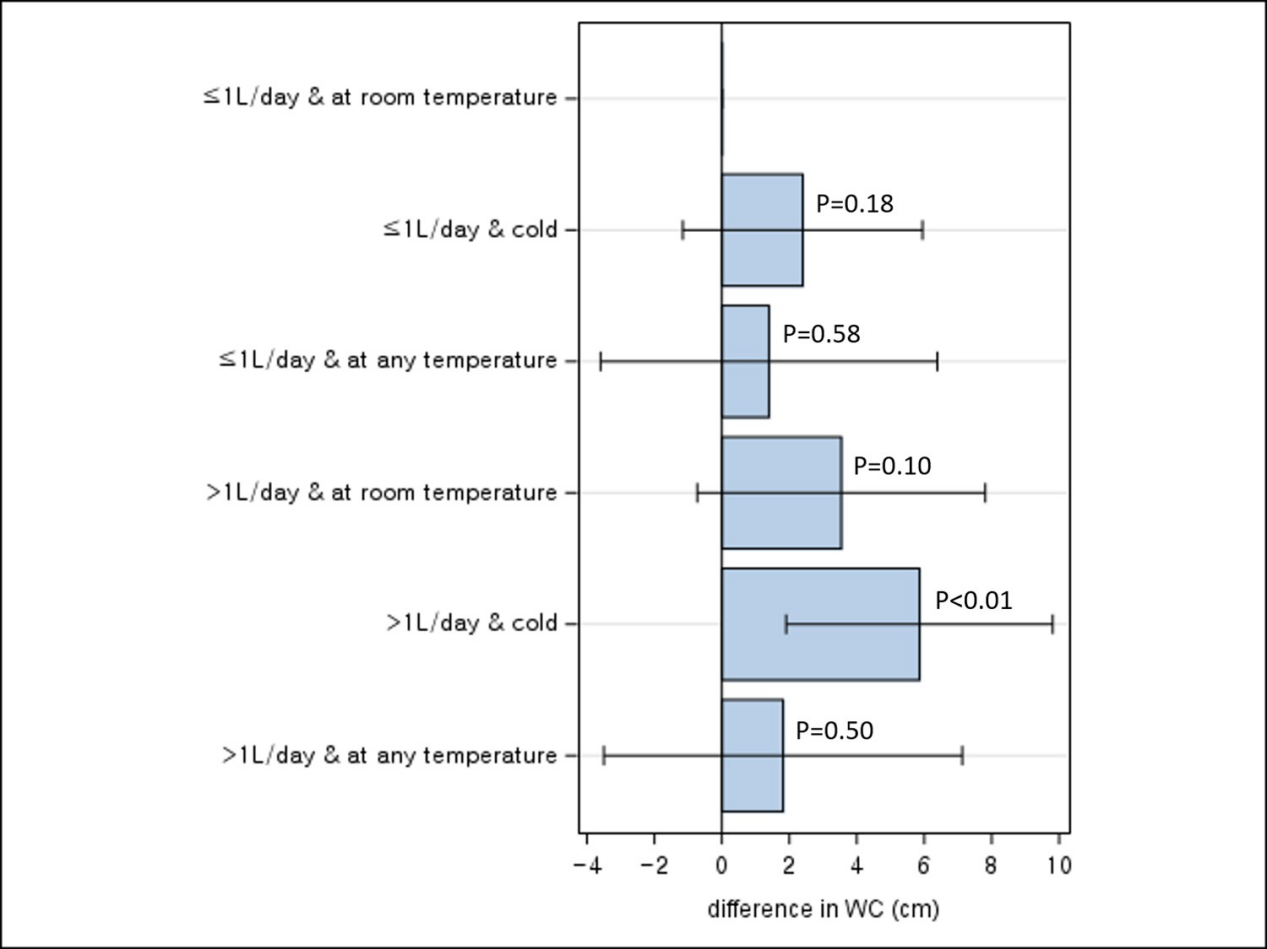
Joint association between total daily water intake and WC according to perceived water temperature.

With WC, a significant increase was observed only for participants drinking >1L/day of water and preferentially cold water (mean difference: 5.86 kg/m^2^, 95% CI 1.91, 9.81), when compared to those drinking ≤1L/day of water and preferentially room-temperature water (Fig 2). The interaction between amount and perceived temperature of drinking water on WC was not significant (P for interaction = 0.63).

### Associations between water intake and adiposity stratified by genetic preposition to obesity

**Fig 3:**
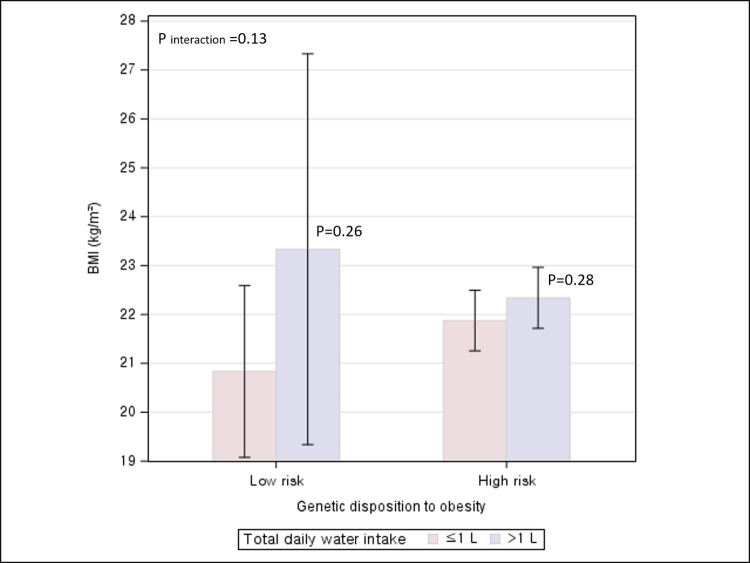
Association between total daily water intake and BMI according to genetic disposition to obesity.

**Fig 4:**
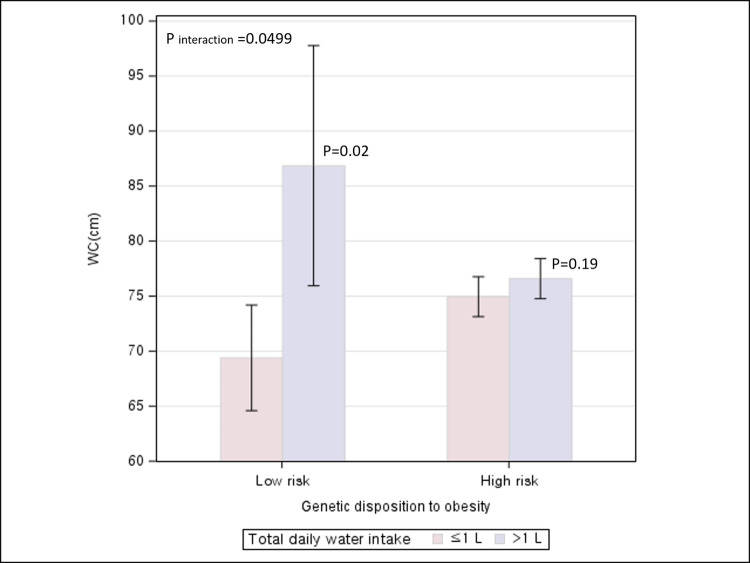
Association between total daily water intake and WC according to genetic disposition to obesity.

An association between total daily water intake and BMI was not significant regardless of low or high genetic prevalence of obesity. (P for interaction = 0.13) (Fig 3). In contrast, the relationship between total daily water intake and WC was significantly differential/modified by genetic predisposition to obesity (P for interaction = 0.0499) (Fig 4). A significant increase in WC associated with drinking >1L/day relative to ≤1L/day was observed among participants with low genetic risk of obesity, but not among those with the high genetic risk.

## Discussion

In this cross-sectional study of 172 participants, drinking >1L/day of water was significantly associated with an increased adiposity as measured by BMI and WC when compared to drinking ≤1L/day. By timing of water intake, drinking water before bedtime was significantly associated with lower BMI, independent of total daily water intake. When perceived temperature of drinking water was considered, significant increases in BMI and WC were most pronounced for drinking >1L/day of water and preferentially cold water when compared to drinking ≤1L/day of water and preferentially warm/room-temperature water. When participants were classified by genetic risk to obesity, a significant positive association between drinking >1L/day and WC was observed among those with low genetic risk of obesity, but not among those with high genetic predisposition.

Consistent with our finding of a positive association between water intake and adiposity, another cross-sectional study of an approximately 8000 Iranian adults showed that individuals who drank >8 glasses/day of water had 1.78-fold greater odds of obesity compared with those who drank <2 glasses/day [22]. Challenging the ubiquitous suggestion that we should drink eight glasses of water a day, a recent study suggested that the widely recommended daily intake of approximately 2L of water, which underestimates water from food, may be excessive for the majority of individuals in most circumstances [23]. In contrast, an inverse association between water intake and adiposity was found in cohort studies of adults free of overweight at baseline in China [24] and overweight young female adults in India [25] and in randomized clinical trials of obese individuals [26, 27]. The discrepant findings across studies might be attributable to multiple factors. First, because a cross-sectional study measures the exposure and the outcome at the same time without a follow-up, reverse causality arising from obese people trying to drink more water as a weight loss strategy might explain the observed positive associations between water intake and adiposity. However, in our study, a higher adiposity associated with a greater water intake was observed among individuals with a lower BMI and WC group as well as those with a higher BMI or WC, which rejects reverse causality as a potential explanation. Second, for the results of clinical trials, albeit least prone to biases, as obese individuals with the goal of weight loss participated in the study, the findings may not be generalizable to the general population with a wide range of weight status. Third, different genetic predisposition to obesity across study populations might be a potential explanation for the inconsistent finding. In our study, a higher WC associated with a greater water intake was observed among individuals with a low genetic risk of obesity but not among individuals with a high genetic risk. This suggests that water, if a causal factor, might be weakly implicated in the development of obesity and thus, its modest effect might be masked given a high baseline risk of obesity conferred by genetic predisposition to obesity or other strong risk factors.

With regard to the timing of water intake, we observed that drinking water before bedtime was inversely associated with BMI, independent of the amount of total daily water intake. The underlying mechanisms are unclear, but the effect of water on the regulation of blood circulation may be implicated. Evidence suggests that moisture loss during sleeping could lead to an increase in the blood viscosity [28]. In a previous study, a high blood viscosity was suggested to be a risk factor of obesity [29, 30]. Drinking water before bedtime may help to keep the body hydrated through the night and to increase blood circulation.

Water intake within 30 minutes before or after a meal was not associated with the adiposity in our study. This finding is in agreement with previous studies showing that pre- or post-meal water intake was not associated with total energy intake, a predictor of obesity risk [3, 31]. For intra-meal water intake, no epidemiologic studies have examined its effect on obesity. Despite the widespread, but scientifically unsubstantiated, claim that water intake while eating harms digestion due to dilution of stomach acids and digestive enzymes, we did not find an association between intra-meal water intake and adiposity.

To our knowledge, this is the first study that analyzed the effect of perceived water temperature on adiposity while accounting for the amount of water intake. While drinking cold water has been widely promoted as a weight loss strategy on the basis that it induces the body to burn calories to heat up the water for the maintenance of a homeostatic internal body temperature [32], in our study, participants who drank preferentially cold water was associated with a higher BMI and WC. This suggests that the effect of cold water intake in boosting energy expenditure may not be sufficient enough to make a meaningful difference in body weight (about 7 kcal over 90minutes when drinking 1L of water at room temperature) [33] and other adverse effects associated with cold water intake, such as restriction of digestion and blood flow due to contraction of muscle and blood vessels and weakened immunity, may outweigh [34–36].

Genetic factors including SNPs have been widely acknowledged for their influence on obesity [37]. The FTO gene (on chromosome 16q12.2), MC4R gene (on chromosome 18q21.3), and BDNF gene (on chromosome 11p14.1) are highly expressed in the brain regions, encoding proteins involved in the regulation of appetite and energy balance [38–40]. SNPs in or near these genes (rs9939609 for FTO, rs17782313 for MC4R, rs6265 for BDNF) are associated increased appetite or energy intake, through which the SNPs are suggested to increase the risk of obesity [41–45]. While no studies have directly linked these genes or SNPs to water intake, higher water intake was shown to decrease energy intake [46–48]. Thus, it is conceivable that these SNPs may interact with water intake in regulating energy intake. In our study, a significant interaction between water intake and genetic predisposition was observed in influencing central adiposity as measured by WC, but not by BMI. If this finding is true, it suggests that the interaction effect might be more pronounced for central adiposity than for overall adiposity. Nevertheless, given a small number of study participants (<200 individuals), the inconsistent findings by measures of adiposity might be due to low power or due to chance.

There are several limitations to this study. First, as a cross-sectional study, the temporal relationship between water intake and adiposity cannot be established and thus, we cannot rule out the possibility that observed associations in our study might be a spurious association driven by reverse causation. While we assessed water intake and anthropometric measurements at the same time when participants visited the research lab at baseline, the questionnaire inquired about average water intake in the past year rather than current water intake, which helps mitigate the degree of reverse causality. Furthermore, as our study does not include obese individuals who are likely to try diverse weight loss methods including increasing water intake, the chance of reverse causality biasing the results is less likely. Second, given that information on water intake was collected through a questionnaire by self-report of participants, the potential for measurement errors in assessing quantity and timing of water intake and perceptions of water temperature is noteworthy. Nevertheless, as the information was collected before the assessment of body weight and waist circumference, errors are likely random with respect to outcome and thus, recall bias is unlikely. Furthermore, as the questionnaire inquired about average water intake over the past year, our analyses reflected participants’ long-term habit accounting for day-to day or seasonal variation in water intake. Third, despite our efforts to adjust for expected potential confounders, there might still remain residual and unmeasured confounding. Fourth, due to small number of obese participants in our study, we were not able to run logistic regression for exploring potential interactions of water amount with perceived water temperature or genetic predisposition to obesity. However, in light of our observation that an association between water amount and obesity was stronger when obesity was analyzed as a categorical variable than as a continuous variable, strengthening of evidence might have observed for interaction analyses on logistic regression if given a sufficient number of participants. Finally, because our participants consist of young healthy Korean adults with a limited range of BMI (15.9–29.5 kg/m^2^) and WC (58–100 cm), our finding may not be generalized to elderly obese adults with high prevalence of strong risk factors of obesity.

Despite the aforementioned limitations, our study is the first that comprehensively investigated the effect of water intake on adiposity in terms of amount, timing, and perceived temperature of drinking water based on a questionnaire specifically designed to address the health effects of water intake, and found consistent results across different measures of adiposity (i.e., BMI and WC). Therefore, our findings help clarify widespread and inconsistent claims on healthy ways to drink water. Furthermore, by performing an analysis based on genetic risk stratification of individuals, our study attempted to provide scientific evidence for individualized recommendation on water intake for weight control.

In conclusion, our study suggests that amount, timing, and perceived temperature of water intake may be implicated in the development of adiposity among healthy young adults. To facilitate weight management, strategies such as moderate (≤1L/day) water intake, avoiding perceived cold water, and encouraging pre-bedtime hydration may be advantageous. For individuals with a low genetic risk of obesity by FTO, MC4R and BDNF, the effect of water intake on abdominal adiposity may be more pronounced. However, considering the cross-sectional nature of our study design based on a small number of participants, cohort studies or randomized controlled trials including a large number of participants are warranted to confirm our findings on diverse aspects of water intake influencing adiposity risk.

## Supporting information

S1 Fig Association between total daily water intake and BMI according to median of BMI. (TIF)

S2 Fig Association between total daily water intake and WC according to median of WC. (TIF)

S1 Table Logistic regression coefficients for the associations between water intake and obesity by timing of water intake. Abbreviation: BMI, body mass index; WC, Waist Circumference. * adjusted for age, sex, genetic predisposition to obesity, perceived water temperature, physical activity, perceived sleep deprivation, perceived stress level, smoking history, alcohol intake, sugar-sweetened beverage intake, breakfast intake, portion size of dinner, night-time snack intake. ** additionally adjusted for total daily water intake; water intake immediately after waking-up and water intake before bedtime were mutually adjusted for. *** additionally adjusted for total daily water intake; pre-meal, intra-meal, and post-meal water intake were mutually adjusted for.
(DOCX)
