# Supplementary figures and images for: Water intake and obesity: By amount, timing, and perceived temperature of drinking water
Source: PLoS One. 2024 Apr 25;19(4):e0301373. doi: 10.1371/journal.pone.0301373 (PMC11045127; doi:10.1371/journal.pone.0301373)

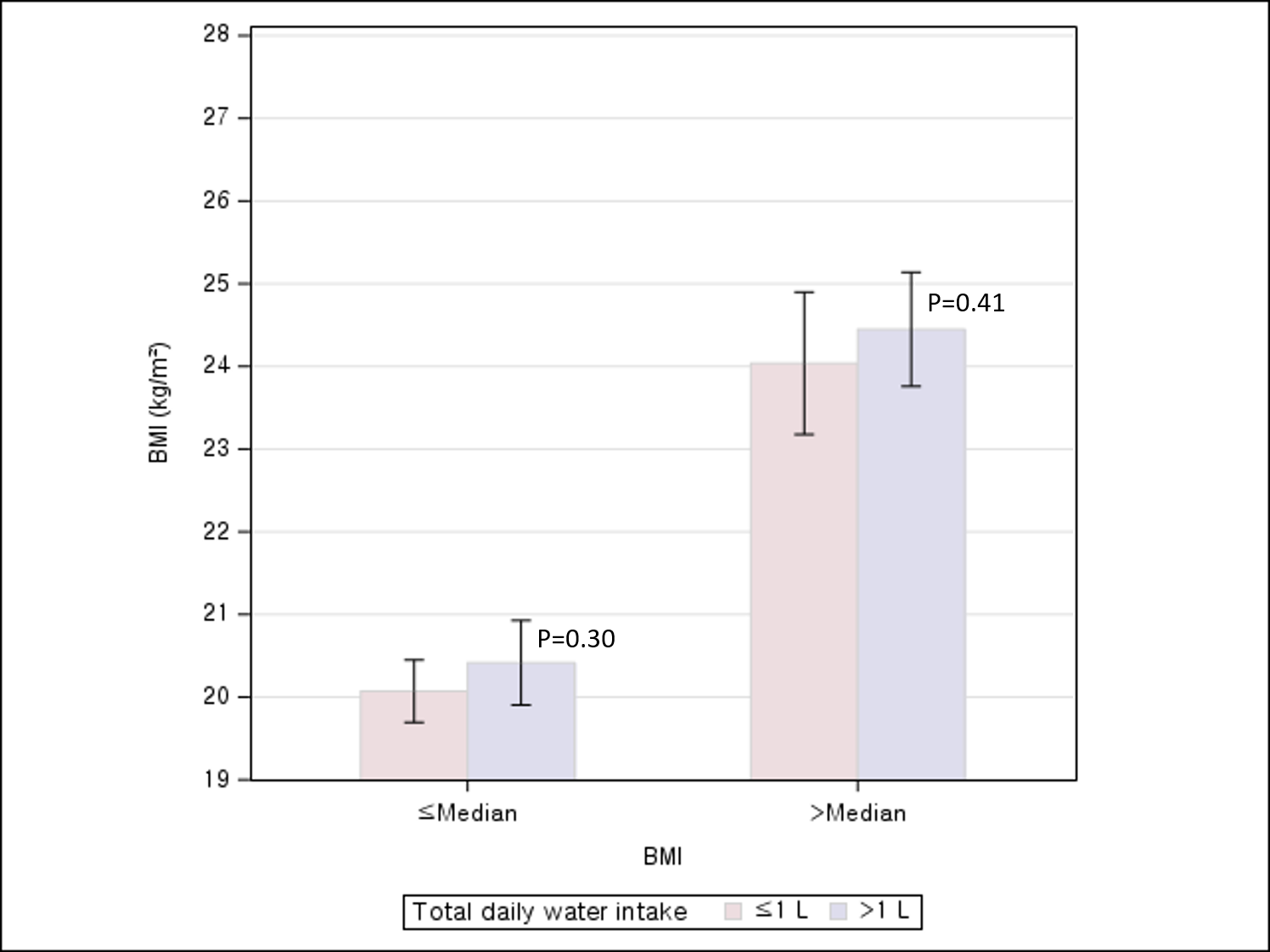

Supplement: S1 Fig — (TIF) [file pone.0301373.s001.tif]

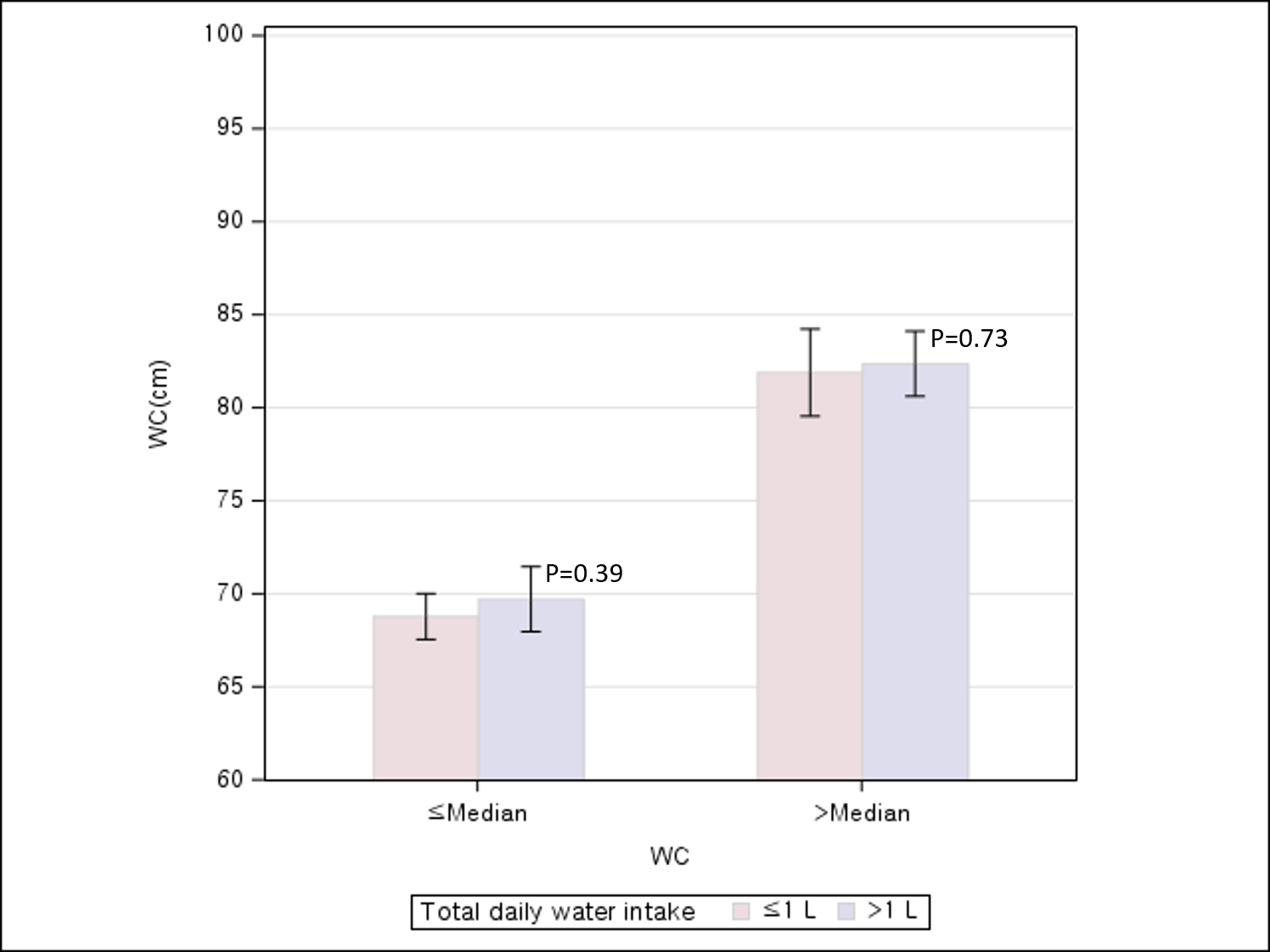

Supplement: S2 Fig — (TIF) [file pone.0301373.s002.tif]
