# Supplementary material for: Water intake and obesity: By amount, timing, and perceived temperature of drinking water
Source: PLoS One. 2024 Apr 25;19(4):e0301373. doi: 10.1371/journal.pone.0301373 (PMC11045127; doi:10.1371/journal.pone.0301373)
Supplement: S1 Table — Abbreviation: BMI, body mass index; WC, Waist Circumference. * adjusted for age, sex, genetic predisposition to obesity, perceived water temperature, physical activity, perceived sleep deprivation, perceived stress level, smoking history, alcohol intake, sugar-sweetened beverage intake, breakfast intake, portion size of dinner, night-time snack intake. ** additionally adjusted for total daily water intake; water intake immediately after waking-up and water intake before bedtime were mutually adjusted for. *** additionally adjusted for total daily water intake; pre-meal, intra-meal, and post-meal water intake were mutually adjusted for. (DOCX) [file pone.0301373.s003.docx]

**S1 Table. Logistic regression coefficients for the associations between water intake and obesity by timing of water intake**

|  | Obesity defined by BMI | | Obesity defined by WC | |
| --- | --- | --- | --- | --- |
|  | Univariable  (95% CI)  P-value | Multivariable*  (95% CI)  P-value | Univariable  (95% CI)  P-value | Multivariable*  (95% CI)  P-value |
| Total daily water intake (>1 vs. ≤1 L/day) | 3.52 (1.84, 6.73),  <0.01 | 2.64 (1.17, 5.93),  0.02 | 2.78 (1.00, 7.70),  0.049 | 3.94 (1.16, 13.33),  0.03 |
| Drinking water immediately after waking up in the morning  (Yes vs. No)** | 1.00 (0.44, 2.29),  >0.99 | 1.03 (0.40, 2.69)  0.95 | 0.67 (0.19, 2.41),  0.54 | 1.02 (0.24, 4.38),  0.98 |
| Drinking water before bedtime (Yes vs. No)** | 0.52 (0.23, 1.19),  0.12 | 0.37 (0.14, 1.00)  0.0496 | 0.21 (0.07, 0.67),  0.01 | 0.19 (0.05, 0.77),  0.01 |
| Pre-meal water intake (≥1 vs. <1 cup/meal)*** | 1.18 (0.51, 2.77),  0.70 | 0.70 (0.25, 2.00),  0.51 | 1.08 (0.33, 3.59)  0.90 | 1.49 (0.39, 5.67),  0.56 |
| Intra-meal water intake (≥1 vs. <1 cup/meal)*** | - 1. (0.51, 1.99),   0.98 | 1.18 (0.51, 2.73),  0.70 | 0.83 (0.29, 2.34),  0.72 | 0.84 (0.26, 2.76),  0.77 |
| Post-meal water intake (≥1 vs. <1 cup/meal)*** | 0.95 (0.46, 1.92),  0.88 | 0.86 (0.36, 2.08),  0.74 | 0.69 (0.23, 2.03),  0.50 | 0.80 (0.24, 2.75),  0.73 |

Abbreviation: BMI, body mass index; WC, Waist Circumference

* adjusted for age, sex, genetic predisposition to obesity, water temperature, physical activity, perceived sleep deprivation, perceived stress level, smoking history, alcohol intake, sugar-sweetened beverage intake, breakfast intake, portion size of dinner, night-time snack intake.

** additionally adjusted for total daily water intake; water intake immediately after waking-up and water intake before bedtime were mutually adjusted for

*** additionally adjusted for total daily water intake; pre-meal, intra-meal, and post-meal water intake were mutually adjusted for
